# Limitation of the therapeutic effort: ethical and legal justification for withholding and/or withdrawing life sustaining treatments

**DOI:** 10.1186/s40248-015-0001-8

**Published:** 2015-02-17

**Authors:** Patrizia Borsellino

**Affiliations:** Philosophy of Law and Bioethics, University of Milano-Bicocca, Milan, Italy

**Keywords:** Decision-making, Deontology, Law, Life sustaining treatments, Limitation of the therapeutic effort, Morals

## Abstract

Withholding and withdrawing a treatment already established are two forms of limitation of the therapeutic effort (LTE). The question of undergoing or not undergoing lifesaving medical treatments is not restricted to a specific health care context, as it refers to a variety of treatments, and it does not concern a restricted group of diseases. LTE has become part of the options compatible with the good clinical practice, in accordance with a deep change in modern medicine’s ‘mission’ along with the increased importance attributed to the patient’s general, personal condition, and to the quality of his/her life. However, LTE remains a controversial issue, and it still has many opponents, in particular, but not exclusively, in those cases in which the question is the withdrawal of treatments, which a widespread conventional wisdom considers ethically and legally different from not initiating treatments. But is it justified to address LTE as a totally controversial issue, especially in case of withdrawal of treatments? The paper answers negatively by arguing that there are criteria both in medical ethics and in law often adequate enough to remove doubts, and to guide decisions and actions.

## Background

Withholding and withdrawing a treatment already established are two forms of limitation of the therapeutic effort (LTE) which consist in not applying pharmacological therapies or instrumental procedures, more or less invasive, considered (by the scientific community) adequate to treat diseases which put life at risk. LTE, framed within the question ‘to what extent and under which conditions it is justifiable to decide not to initiate a treatment or to withdraw it in case it has already been established’, presupposes a wide availability of lifesaving therapeutic tools. Therefore, LTE is strictly connected with the extraordinary advances in modern technology and medicine, occurred in the second half of the XX century. Since then, modern medicine has constantly increased its power to control death by modulating its duration and modes, as it disposes of procedures to prolong survival termed ‘lifesaving medical treatments’. This term refers to a wide range of interventions and treatments including not only resuscitation practices and mechanical cardiopulmonary ventilation, but also those procedures useful to support or replace basic compromised vital functions, as in the case of dialysis in case of kidney dysfunction, or of devices like defibrillators helpful to contrast lethal cardiac dysfunctions. But the prevalent opinion accepted by the majority of the scientific community also includes artificial nutrition and hydration, along with many categories of pharmacological treatments like antibiotics. Consequently, the question of undergoing or not undergoing lifesaving medical treatments is not limited to a specific health care context, as it refers to a variety of treatments, and it does not concern a restricted group of diseases. On the contrary, LTE is a comprehensive problem. Not only physicians dealing with resuscitation and intensive therapies face it, but also those who take care of terminal patients - often suffering from a polypathological condition - with chronic degenerative diseases (oncological, neurological, cardiovascular, kidney diseases), as well as those physicians who work with a growing aging population with serious forms of cognitive impairment due to Alzheimer’s disease or other forms of senile dementia. Therefore, the first but not unique prerequisite concerning LTE is the great availability of tools useful to prolong survival. This represents a distinguishing feature of modern medicine if compared to the pre-modern one, which faced the opposite problem, i.e. the scarcity of therapeutic tools. The further, not less relevant condition is the ‘problematization’ of the assumption that each therapy, in particular if lifesaving, has to be considered useful, and as such deserving to be applied in any case to the patient as it is available. In other words, the question if everything we can do to keep an individual alive ought also to be done, presupposes that the ‘therapeutic privilege’ is brought into question. According to this ‘privilege’, the physician has traditionally had the exclusive power to decide if to administer a treatment, and if to intervene to restore health, or at least and in any case, to choose all those interventions needed to safeguard patients’ survival.

Today, LTE has become part of the options compatible with the good clinical practice, in accordance with a deep change of the modern medicine’s ‘mission’ along with the increased importance attributed to the patient’s general, personal condition, and to the quality of his/her life.

However, LTE remains a controversial issue, and it still has many opponents, in particular, but not exclusively, in those cases in which the question is the withdrawal of treatments, which widespread conventional wisdom considers ethically and legally different from not initiating them. But is it justified to address LTE as a totally controversial issue, especially in case of withdrawal of treatments? The paper answers negatively by arguing that there are criteria both in medical ethics and in law often adequate enough to remove doubts, and to guide decisions and actions.

## Discussion

### LTE and refusal of treatments in case of competent patients

The framework, within which the issue of LTE has to be addressed, is the one defined by those principles and rules, which govern both the international and national legal order, along with the context of medical ethics. Those principles and rules have affirmed the key role played by the self-determination principle in making treatment decisions. If it can be stated that patients’ self-determination in Anglo-Saxon countries finds a culturally more fertile ground than in countries with dissimilar traditions, for instance the New-Latin ones like Italy, it cannot be denied that, even in Italy, medical practice is subjected to rules established at different levels of the legal order ^a^ and to provisions stated both in the Code of Medical Ethics (2006), and in the Code of Ethics for Nurses (2009). All these rules link the power/duty of care to the respect of the patient’s will, which the patient expresses by accepting through informed consent or by dissenting, and therefore refusing a given treatment.

The current legal-ethical framework subordinates all medical interventions and treatments to the patient’s will, without providing exceptions either in relation to patients in critical conditions or to lifesaving treatments. This system affects the whole system of physicians’ powers, duties, and liabilities, and generally of health care workers. Moreover, it also influences the question of LTE directly. Indeed, from the mentioned legal-ethical framework follows that not all measures with therapeutic purposes and applied in respect of the rules of the art, but even not all measures, which, if not initiated, put the patient’s life at risk, ought to be considered legitimate, or even owed. Given this legal-ethical framework, we can regard those measures to which the patient (also in emergency cases) has consciously consented after being adequately informed about the nature of the intervention and the risks deriving from not undergoing it as legitimate.

It follows that if a competent patient refuses a life sustaining treatment, which is considered too burdensome or incompatible with his/her beliefs, the physician has the duty to desist from administering it^b^, by not including it in the available options. However, withdrawal of treatments does not equate with withdrawal of care, achieved by establishing palliative measures to relieve suffering and pain. The patient’s firm and unmistakable refusal of a lifesaving treatment impacts on the physician’s commitment to preserving survival at any cost. Differently from the past, when the physician’s commitment to preserving survival at any cost was considered ‘a mandatory content’ of the physician’s responsibility toward his/her patient [1]. Instead today, there is no doubt that the patient’s valid consent or refusal is fundamental in terms of lawfulness and imperativeness to the decision of initiating or not initiating a treatment. This is true even in those cases in which this could prevent death or imply stopping a treatment already established, and in some cases administered to the patient for a long time.

This occurred in the case of Piergiorgio Welby^c^ [1-3], who made headlines in Italy some years ago. This case represented a paradigmatic example of a wider case history in the clinical practice. When the patient dissents from maintaining the administered life sustaining treatment - in Welby’s case assisted ventilation - though originally accepted, the treatment is no longer legitimate. Indeed, its refusal undermines its ethical and legal foundation, in just the same way as the lack of a valid consent results in an illegitimate and morally unacceptable administration of a treatment refused by the patient.

### LTE in case of patients lacking decision-making capacity: the advance directives’ way

The will of the patient able to make decisions offers a clear and well-defined criterion to guide decisions with regard to LTE in both above-mentioned forms. Nevertheless, this criterion is still undervalued and not sufficiently implemented, on the one hand, by reason of the persistence among health workers of attitudes and approaches not faithful to the mandatory duty of information and of patient’s participation in the decision-making process. On the other hand, psychological resistance to confrontation with death still plays a role in maintaining the rooted conviction that death represents the physician’s burning failure, to be avoided by any means.

But what is to be done in those numerous situations in which patients lack decision-making capacity?

The first aspect to be underlined is that, with regard to this specific health care context, which has been traditionally considered subject to the physician’s exclusive decisional power - and to some extent it still is - the new, guiding, clinical decision-making model recognizes the central role played by the patient’s will, beliefs, and values, and by his/her participation – though indirectly to health care decisions.

This purpose can be achieved by taking into account the patient’s will expressed before becoming incompetent with regard to the treatments he/she accepts to undergo or not in relation to future pathological conditions (living will), and contextually, or as an alternative, with reference to the appointment of an agent (substitute person) to make his/her health care decisions and to participate to the decision-making process (health care power of attorney). This is the way of the advance directives, for which there is no legal regulation in Italy, different from other European and extra-European countries [4], despite the numerous bills [1] elaborated by the Italian Parliament starting in 2004. However, in spite of this situation, there is no legal vacuum. Indeed, there are legal and ethical rules which oblige the physician to take into account previously expressed wishes^d^, but even prior to these, there are provisions of the Italian Constitution which prohibit unjustified unequal treatments due to personal conditions (Art. 3), and protect the fundamental freedom of thought (Art. 21) and of religion (Art. 19).

In principle, the widespread practice to subscribe advance directives may supply physicians with a formal expression of the patients’ will-in some cases, in favor of withholding or withdrawing life sustaining treatments - also in those situations in which the loss of competence is due to accidental causes or to a sudden event. However, in the majority of the cases, the loss of competence follows the development of a degenerative disease, or it is induced as it occurs for surgical interventions performed under general anesthesia. And these are cases for which it is realistic and hopeful to include the practice of subscribing advance directives in the LTE’s criteria. In the circumstances envisaged, in which decisions concern future events not radically undefined, collecting the patient’s expressed wishes within an advance care planning program represents the ethically correct strategy to promote the perfect extension of the impacts of self-determination to patients lacking decision-making capacity [5]. In this way, it would be possible to avoid the risk of inappropriate treatments with regard to objectives in terms of health and quality of life, which no one can presume to evaluate better than the patient himself/herself.

### LTE between medical evaluation and substitute decisions

The appreciation of the patient’s advance as well as current will is required by the model of therapeutic relationship inspired by the idea that the duplicity of those subjects involved in the clinical decision-making process should not be lost. And this is true even in those cases in which decisions concern incompetent patients, and even more so when critical decisions are made, for instance withholding or withdrawing lifesaving treatments. In the Italian legal framework, though in absence of a legal act regulating the powers of the trustee designated by the beneficiary of the health care in a ‘living will’, the above-mentioned model has been promptly implemented by those legal rules providing figures like guardians^e^ and ‘supporting administrators’^f^, who are responsible for the care of the person and have the task to interact with the health care workers, because it is stated that, when a person “…does not have the capacity to consent to an intervention,…[this] intervention may only be carried out with the authorization of his or her representative or an authority or a person or body provided for by law”^g^.

On the one hand, following the opposite trend to the traditional paternalistic approach, this model no longer permits regarding the physician as the unique legitimate decision-maker. On the other hand, however, the same model demands both giving proper consideration to the role played by the medical evaluation and not misinterpreting the nature and limits of the decision power given to the person appointed.

As for the medical evaluation, the main aspect to be underlined is that, in any pathological condition, irrespective of the patient’s capacity or incapacity to make valid decisions, it plays a fundamental role in identifying those treatments to be considered adequate, and therefore worthy of proposing. Indeed, the medical evaluation, which alone can define the foreseeable scenarios in relation to administering selected treatments or not, is the premise of any expression of the patient’s conscious will, including the refusal of a lifesaving treatment already established or to be initiated. In any case, there is no doubt that the medical evaluation, grounded on evidence of clinical effectiveness, earns prime relevance when it is impossible to refer to the patient’s current or advance expressed will. This occurs in the majority of emergency interventions on incompetent patients due to accidental causes (paradigmatic examples are interventions in the ER on victims of car accidents), and in the circumstances envisaged, there is no place for legal representatives, whose substitute authorization is generally provided for interventions on incompetent patients by legal and ethical rules. In these situations, as it occurs in other numerous cases, in which the unavailability of the patient’s will does not depend on the circumstances, rather on the enduring resistance to inform him/her and to make an advance care planning, it is up to the physician to evaluate if and on what conditions a treatment functional to prolonging survival is also adequate, and therefore, if and to what extent it has to be administered or not. However, what are useful standards for defining the inadequacy on which it is possible to justify withholding or withdrawing treatments according to clinical evaluation? In the United States, where the patients’ self-determination has been emphasized earlier and more than in other countries, the question has been addressed - in the late 80s and early 90s - as part of the debate on the ‘medical futility’. In this frame, it was initially argued that useless treatments, i.e. “that cannot reasonably be expected to achieve even its physiological objective”^h^, ought not to be administered. This initial restrictive interpretation, which seems to limit the evaluation concerning the inadequacy of a treatment to the meagerness of the advantage in terms of quantity of life, and therefore to the predictability of its failure, has been overcome by a more comprehensive trend, which has been confirmed by provisions in relevant international documents along with important stances made on the part of Italian scientific associations^i^. On the one hand, supporters of this new trend are in favor of the inclusion, within the criteria which justify LTE, of treatments’ inadequacy with regard to the objective of achieving a significant benefit and of improving the patient’s quality of life. And on the other hand, they consider the treatments’ onerousness as part of LTE’s criteria. This onerousness is viewed from a psychological perspective when referred to the patient’s family members, and from a stricter economic viewpoint when it concerns society as a whole. The inclusion of a society-related viewpoint in the LTE’s criteria is justified by the consideration that the administration of treatments to patients who cannot benefit from them because of the seriousness of their condition deprives society of precious resources, which could be employed to treat patients with a different clinical situation.

Therefore, LTE, which can be considered justifiable, above all on the ground of the medical evaluation, is the one which aims at achieving the best interest of the patient, without ignoring other interests at stake. The definition of the ‘best interest’ takes into account the peculiarity of the patient’s clinical situation along with his/her expectations. These are exactly the main factors to be considered by the substitute decision-maker. Indeed, when a person, other than the recipient of the treatments, is involved in the decision-making process, he/she does not have the unconditional power to dispose of the incapable patient’s health and life. His/her powers are restricted by the ‘best interest’ of the beneficiary. This ‘best interest’ could be achieved if one decides neither ‘in place of’ nor ‘for’ the incapable person, but ‘with’ him/her, as the Italian Court of Cassation effectively stated in its decision on the Englaro case^j^. In other words, the decision is made in accordance with the patient’s will, and when this is presumed, by inferring it from his/her personality, life style, inclinations, and moral values.

## Conclusions

Life sustaining treatments –ranging from resuscitation techniques, assisted ventilation, to artificial nutrition and hydration - represent valued and irreplaceable devices in the context of modern medicine. However, we should not lose sight of their aim, which consists in recovering vital compromised functions, or at least in controlling suffering and in maintaining a level of quality and dignity of life which the patient considers acceptable, and not in prolonging the death process of individuals who cannot benefit from lifesaving treatments because of their critical health state. With regard to these patients, health care workers are not always morally, deontologically, and legally obliged to initiate or to continue lifesaving treatments. Instead, LTE may rather represent the needed premise to give the kind of assistance, in the palliative care’s perspective, whose main purpose is to preserve ill persons from unnecessary suffering, granting them their dignity until the end of life.

As far as the withdrawal of a life sustaining treatment already established is concerned, the persistence of the ‘prejudice’ about its generalized illegality will hinder the activity of a good clinical practice. Instead, it would be of great advantage for a good practice the spreading and rooting of the idea that, in uncertain situations, it could be worth initiating a treatment, which could benefit the patient, rather than abandoning the option of administering it in fear of the impossibility of withholding it once initiated.

## Endnotes

^a^Articles 13 and 32 of the Italian Constitution; Article 5 of the Convention on Biomedicine and Human Rights (Oviedo 1997); Article 3 of the Charter of Fundamental Rights of the European Union (Nizza 2000). Both the Convention on Biomedicine and Human Rights and the Charter of Fundamental Rights of the European Union are part of the highest level of the Italian legal order since the Lisbon Treaty entered into force in 2009.

^b^The Code of Medical Ethics (2006), Article 35 “the physician cannot perform any diagnostic or therapeutic treatment without the patient’s explicit and informed consent… In any case, in the presence of a recorded refusal of a capable person, the physician must desist from the consequent diagnostic and therapeutic acts as it is prohibited any medical treatment administered against the patient’s will”.

^c^Welby was diagnosed with muscular dystrophy as a teenager in the early 1960s. The disease progressed, and in 1997 he became unable to breathe on his own. However, he was capable of expressing consent or refusal to medical treatments and did not need a third person as his legal representative. Therefore, he asked his physician to discontinue mechanical ventilation and meanwhile to administer him palliative sedation. For a long time his request to discontinue medical treatments has remained unheard. The main reason for not complying with his request was the risk for the physician of being indicated for murder (Article 575 of the Italian Criminal Code. In Italy, as there is no Act concerning euthanasia and/or assisted suicide, the courts apply the provisions of the criminal code concerning murder). The physician who eventually stopped the mechanical ventilation was later tried and acquitted of a ‘killing on demand’ charge (‘Killing on demand’, that is ‘omicidio del consenziente’, takes place when the person expresses a consent to be killed. Decision n. 2047/2007 of the Court of Rome). Indeed, in this case, as Welby's request was voluntary and not dictated by external pressures, there were no ethical and legal arguments for impeding the application of the principle of ‘willingness’ to treatments (Article 32 Italian Constitution).

^d^Convention on Biomedicine and Human Rights (Oviedo 1997), Article 9; the Code of Medical Ethics (2006), Articles 16, 35, 36, 38.

^e^See Article 357 of the Italian Civil Code concerning personal care decisions on the part of the legal representative.

^f^The’supporting administrator’ has been introduced in Italy with Act 6, 9 January 2004, which reformed Articles 404–414 of the Italian Civil Code.

^g^Convention on Biomedicine and Human Rights, Article 6.3.

^h^*The Appleton Consensus,* 1992, in *Bioetica. Rivista interdisciplinare*, 1992, 3, pp. 397–431.

^i^See two documents of the Italian Bioethics Commission of the Italian Society of Anesthesia, Analgesia, and Intensive Care (SIAARTI),), *Raccomandazioni SIAARTI per l‘ammissione e la dimissione dalla terapia intensiva e per la limitazione dei trattamenti in terapia intensiva*, in *Minerva anestesiologica*, 69, 2003, pp. 101–118 e *Le cure di fine vita e l’anestesista rianimatore: Raccomandazioni SIAARTI per l’approccio al malato morente*, in *Minerva anestesiologica*, 72, 2006, pp.1-23.

^j^Court of Cassation, Civil Section. Decision n. 21748, 2007, October 18.

## Abbreviation

LTE: limitation of the therapeutic effort
